# Final 4-year results of the RAINBOW real-world study: intravitreal aflibercept dosing regimens in France in treatment-naïve patients with neovascular age-related macular degeneration

**DOI:** 10.1007/s00417-022-05900-6

**Published:** 2022-11-18

**Authors:** Salomon-Yves Cohen, Marcel Dominguez, Florence Coscas, Céline Faure, Stéphanie Baillif, Hassiba Oubraham, Laurent Kodjikian, Michel Weber, on behalf of the RAINBOW study investigators

**Affiliations:** 1 Centre d’Imagerie et de Laser, Paris, France; 2 Department of Ophthalmology, University Paris-Est Créteil, Paris, France; 3 Centre Rétine Gallien, Bordeaux, France; 4 Centre Ophtalmologique de l’Odéon, Paris, France; 5 Hôpital Privé Saint Martin, Ramsay Générale de Santé, Caen, France; 6 Pasteur 2 Teaching Hospital, Université Côte d’Azur, Nice, France; 7 Centre OPHTA-45, Montargis, France; 8 Croix-Rousse University Hospital, Hospices Civils de Lyon, University of Lyon I, Lyon, France; 9 CNRS, MATEIS INSA Lyon, Université de Lyon Claude Bernard, 510, Villeurbanne, France; 10 CHU Hôtel-Dieu, Nantes, France

**Keywords:** Aflibercept, Intravitreal injections, Neovascular age-related macular degeneration, Real-world study, Long-term treatment outcomes

## Abstract

**Purpose:**

The purpose of this study is to evaluate real-world treatment outcomes in patients with neovascular age-related macular degeneration (nAMD) treated with intravitreal aflibercept (IVT-AFL) in routine clinical practice in France.

**Methods:**

RAINBOW (NCT02279537) was an ambispective, observational, 4-year study assessing IVT-AFL effectiveness, treatment patterns, and safety in patients with nAMD in France. Treatment-naïve patients prescribed IVT-AFL and treated according to local practice (pro re nata or treat-and-extend) were eligible. Three treatment cohorts were retrospectively identified based on their treatment pattern within the first 12 months: regular (3 initial monthly IVT-AFL injections received within 45–90 days after the first injection in month 0 and followed by injections every 2 months), irregular with the initial monthly injections, and irregular without the initial monthly injections. The primary endpoint was mean change in best-corrected visual acuity (BCVA) from baseline to month 12. The 48-month results are described here.

**Results:**

Overall, the study included 516 patients (each with one study eye), and 30.2% of patients completed 48 months of IVT-AFL treatment. Mean change in BCVA from baseline (56.5 letters) to month 48 for patients with an assessment at both time points was + 1.1 (regular cohort, *n* = 47), + 0.1 (irregular cohort with initial monthly injections, *n* = 115), and − 1.3 letters (irregular cohort without initial monthly injections, *n* = 26), representing a decrease from the gains achieved at month 12. Mean number of IVT-AFL injections received by month 48 in the treatment cohorts was 14.9, 13.7, and 11.9, respectively. The safety profile of IVT-AFL was consistent with previous studies.

**Conclusion:**

In RAINBOW, the 48-month results demonstrate a lack of long-term effectiveness of IVT-AFL treatment of nAMD due to progressive undertreatment in routine clinical practice in France. These real-world findings highlight the importance of 3 initial monthly IVT-AFL injections followed by continuous proactive treatment beyond the first year to achieve optimal functional outcomes.

**Trial registration number:**

ClinicalTrials.gov Identifier: NCT02279537.

**Supplementary Information:**

The online version contains supplementary material available at 10.1007/s00417-022-05900-6.


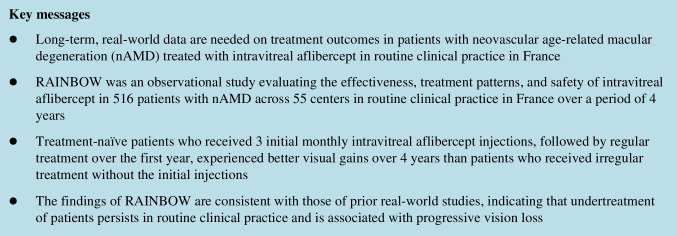


## Introduction

Anti-vascular endothelial growth factor (anti-VEGF) therapies, which include intravitreal aflibercept (IVT-AFL) and ranibizumab, are the standard of care for the treatment of neovascular age-related macular degeneration (nAMD) [1]. Based on the results of the VIEW studies [2, 3], IVT-AFL was approved for use in Europe in 2012 with bimonthly injections after 3 initial monthly injections [4]. This treatment interval may be extended beyond 2 months after the first year if specific visual and anatomic criteria are met.

Visual acuity gains achieved with anti-VEGF therapies in randomized controlled trials (RCTs) are not always realized in real-world clinical settings [5]. RCTs provide a more controlled environment with more selective inclusion criteria than observational studies, which may limit the relevance of findings. In contrast, real-world evidence (RWE) generated in routine clinical practice provides data on effectiveness and treatment patterns in more heterogeneous patient populations. This allows greater insight into factors such as adherence to and persistence with treatment and the effects these factors have on visual outcomes [6]. Thus, generating RWE is valuable to patients and clinicians to better understand and address the discrepancy in outcomes between RCTs and real-world experience.

The focus of most real-world studies of nAMD has been on ranibizumab (because of its earlier European approval in 2007) [7] and the pro re nata regimen of anti-VEGF treatment [6]. In France, RWE has been generated from the retrospective LUMIERE [8], TWIN [9], and AURA [10] observational studies of ranibizumab treatment over 12 months and from a retrospective single-center analysis of 10-year intravitreal anti-VEGF therapy outcomes using Fight Retinal Blindness! (FRB!) data [11]. However, large-scale multicenter observational studies of IVT-AFL in patients with nAMD in France are lacking.

RAINBOW (NCT02279537) was a multicenter observational study evaluating the effectiveness, treatment patterns, and safety of IVT-AFL in treatment-naïve patients with nAMD in routine clinical practice in France over a period of 4 years. The RAINBOW 1-year [12] and 2-year results [13] have previously been published, as has a subgroup analysis of the 1-year results, which compared effectiveness in patients receiving regular and irregular IVT-AFL treatment [14].

Briefly, by month 12 in RAINBOW, the overall mean change in best-corrected visual acuity (BCVA) from baseline was + 5.0 letters (+ 7.1 in the regular cohort, + 5.6 in the irregular cohort with 3 initial monthly injections, and − 1.1 in the irregular cohort without 3 initial monthly injections) [14]. By month 24, the overall mean change in BCVA from baseline was + 3.0 letters (+ 4.9 and + 4.0 letters in the regular and irregular with 3 initial monthly injection cohorts, respectively, and − 2.5 letters in the irregular cohort without 3 initial monthly injections) [13]. Here, we report the final 4-year results of the RAINBOW study. The findings are presented for the overall cohort and stratified according to IVT-AFL treatment pattern.

## Methods

### Study design

A detailed description of the RAINBOW study (NCT02279537) methodology has previously been published [12]. RAINBOW was an ambispective (i.e., containing both retrospective and prospective stages of data collection), observational, 4-year study designed to evaluate the effectiveness, treatment patterns, and safety of IVT-AFL in patients with nAMD in routine clinical practice across 55 centers in France. Data collection was initiated in October 2014, and data from patients who started IVT-AFL treatment between January 2 and October 13, 2014, were retrospectively collected; data were then prospectively collected from October 14, 2014, to April 17, 2019. The ambispective design did not affect the data collection process nor the study methods in any way that would warrant a separate analysis of the retrospectively and prospectively collected data.

### Patients and procedures

Treatment-naïve patients with a diagnosis of nAMD in the study eye and for whom the decision to treat with IVT-AFL had been made were eligible for inclusion. Patients must have received their first IVT-AFL treatment between January 1, 2014, and April 30, 2015. Patients were excluded if they had another retinal disease, did not meet the local indication criteria, or were participating in an interventional study. All decisions regarding treatment, diagnosis, and follow-up were at the discretion of the attending physician according to local medical practice. Patients were treated with the option of a reactive approach (pro re nata) or an individualized proactive approach (treat-and-extend [T&E]), in accordance with the European Medicines Agency Summary of Product Characteristics for IVT-AFL [4].

In patients with both eyes treated but with different treatment start dates for each eye, the first eye to be treated was retained as the study eye. In patients with both eyes treated during the initial visit, the eye with the worst BCVA at baseline was retained as the study eye in the full analysis set (FAS); where the BCVA was similar between both eyes at baseline, the right eye was selected as the study eye by convention. Therefore, patients with both eyes treated were included only once in the FAS. For the safety analysis, both treated eyes (where applicable) were retained in the safety analysis set (SAS).

### Endpoints

The primary endpoint was the mean change in BCVA in Early Treatment Diabetic Retinopathy Study (ETDRS) letters or any visual logarithmic scale from baseline to month 12; these results and a subgroup analysis thereof have previously been published [12, 14]. Results at month 48 according to IVT-AFL treatment pattern are presented here.

Secondary outcomes included the mean change in BCVA from baseline to months 24, 36, and 48; the percentage of patients who experienced a BCVA gain of ≥ 0 letters, ≥ 5 letters, ≥ 10 letters, and ≥ 15 letters from baseline to months 24, 36, and 48; the percentage of patients who maintained vision (defined as a BCVA loss of < 15 letters) at months 24, 36, and 48; and the mean number of injections and visits over the study period. Safety was monitored throughout the study. All adverse events reported after the first IVT-AFL treatment and up to 30 days after the last IVT-AFL treatment were documented as treatment-emergent adverse events.

### Statistical analysis

Based on the VIEW studies [2, 3] and a 10% annual dropout rate, 600 patients had to be enrolled in the RAINBOW study to achieve a minimum sample size of 390 patients at month 48. The FAS comprised all patients who received ≥ 1 IVT-AFL injection and for whom visual acuity and anatomic assessments (in the study eye) had been performed at baseline (≤ 30 days before the first IVT-AFL treatment) and at least once during follow-up. The SAS included all patients who received ≥ 1 IVT-AFL injection.

Although both the pro re nata and T&E treatment approaches include 3 initial monthly injections, not all patients in the RAINBOW study received these initial injections (defined here as the first 3 injections received within 45–90 days after the first injection in month 0 for a total of ≥ 4 injections within the first 90 days). Therefore, patients were retrospectively divided into 3 cohorts depending on their treatment pattern during the first 12 months of treatment. Specifically, the regular cohort comprised patients who received the 3 initial monthly (− 1/ + 2 weeks) IVT-AFL injections followed by IVT-AFL injections every 2 months (− 3/ + 4 weeks). The 2 irregular cohorts included patients who received IVT-AFL injections every < 2 or > 2 months over the first 12 months, with or without the 3 initial monthly (− 1/ + 2 weeks) injections.

All data collected up to month 48 were analyzed for the overall FAS. Data from the FAS population were analyzed on an intent-to-treat basis and included data after any switch from IVT-AFL to another treatment. To analyze data during treatment with IVT-AFL specifically, an additional exploratory analysis was conducted in a population defined as the “FAS before switch;” in this population, data were included until the switch and considered missing after the switch. Outcomes at 4 years are reported here for the overall FAS population, the FAS before switch population, and for patients in the FAS stratified by treatment pattern during the first year.

Statistical analyses were explorative and descriptive; the study did not aim to confirm or reject predefined hypotheses. Continuous variables were described by absolute values and as changes from baseline per analysis time point. All data reported here are for patients with assessments at each of the indicated timepoints, and none of the results are based on a last observation carried forward (LOCF) analysis or any other method of imputation. A mixed model for repeated measures was used to estimate the change in BCVA over time in the FAS before switch population (fixed effects: baseline BCVA and time). In addition, several robustness analyses were performed on the FAS (data not reported here). These analyses included imputation of missing data by replacement with the patient’s last observed value (i.e., using the LOCF approach), imputation of missing data by replacement with the median value of the population, and smoothing imputation. Statistical analyses were performed by use of the SAS software package, release 9.4 (SAS Institute Inc., Cary, NC, USA).

## Results

### Patients

The patient disposition in the RAINBOW study is shown in Online Resource 1, and baseline demographics and disease characteristics are listed in Table 1. Overall, 591 patients were included in the SAS and 516 patients were included in the FAS. There are differences in the number of patients in the FAS and SAS between the present analysis and the RAINBOW 12-month and 24-month analyses. These differences were due to the database not being locked until the end of the study and queries affecting the inclusion of certain patients being resolved between the month 12, month 24, and month 48 analyses.

**Table 1:** Patient baseline demographics and disease characteristics

	Full analysis set (*N* = 516)
Patient characteristics	
Age (years)	79.6 ± 7.9
Female, *n* (%)	317 (61.4)
Mean duration of nAMD (days)^a^	30.5 ± 221.4
Median duration of nAMD (days)^a^	4.0
Visual characteristics	
BCVA, letters	
Overall (*N* = 516)	56.5 ± 18.8
Regular cohort (*n* = 102)	59.0 ± 17.7
Irregular cohort with initial injections (*n* = 253)	56.3 ± 19.0
Irregular cohort without initial injections (*n* = 56)	57.2 ± 20.8
BCVA categories, *n* (%)	
< 50 letters	143 (27.7)
50 to < 55 letters	61 (11.8)
55 to < 70 letters	149 (28.9)
≥ 70 letters	163 (31.6)
Anatomic variables	
CRT (*n* = 464) (μm)	400 ± 141
SRF (*n* = 499), *n* (%)	406 (81.4)
IRF (*n* = 499), *n* (%)	314 (62.9)
PED (*n* = 499), *n* (%)	314 (62.9)
Height of PED (*n* = 314) (μm)	289.2 ± 215.0
Sub-RPE fluid (*n* = 499), *n* (%)	244 (48.9)

Data are mean ± SD unless otherwise indicated. The regular and irregular cohorts include only those patients from the FAS with a BCVA assessment at baseline and month 12. ^a^Duration of nAMD between diagnosis and first treatment. *BCVA* best-corrected visual acuity, *CRT* central retinal thickness, *FAS* full analysis set, *IRF* intraretinal fluid, *nAMD* neovascular age-related macular degeneration, *PED* pigment epithelial detachment, *RPE* retinal pigment epithelium, *SRF* subretinal fluid.

Patients were aged 52–97 years (mean age, 79.6 years), and 61.4% of patients were female (Table 1). At baseline, the mean BCVA was 56.5 letters, and the mean central retinal thickness (CRT) was 400 μm. The median duration between diagnosis and the first IVT-AFL injection was 4 days. In the FAS population, most patients received unilateral treatment (*n* = 447, 86.6%), whereas 69 patients (13.4%) received bilateral treatment.

### Treatment pattern and exposure

Of the 411 of 516 patients in the FAS with a BCVA assessment at months 0 and 12, 102 patients (24.8%) were treated regularly with IVT-AFL and comprised the regular treatment cohort, whereas the remaining 309 patients were treated irregularly. Of the 309 irregularly treated patients, 253 (81.9%) received the 3 initial monthly injections and 56 (18.1%) did not.

In the overall FAS, the mean ± SD duration of treatment with IVT-AFL (defined as the mean time between the first and last IVT-AFL injections) was 29.4 ± 18.2 months, with a median of 32.9 months. Based on Kaplan–Meier estimates, 91.0% of patients in the FAS completed 3 months of IVT-AFL treatment, 75.1% completed 12 months, 55.2% completed 24 months, 45.5% completed 36 months, and 30.2% completed 48 months. Premature discontinuation from the study (*n* = 232) was mainly due to loss to follow-up (*n* = 131 [56.5%]), transfer to another physician (*n* = 39 [16.8%]), and death (*n* = 30 [12.9%]).

**Fig. 1:**
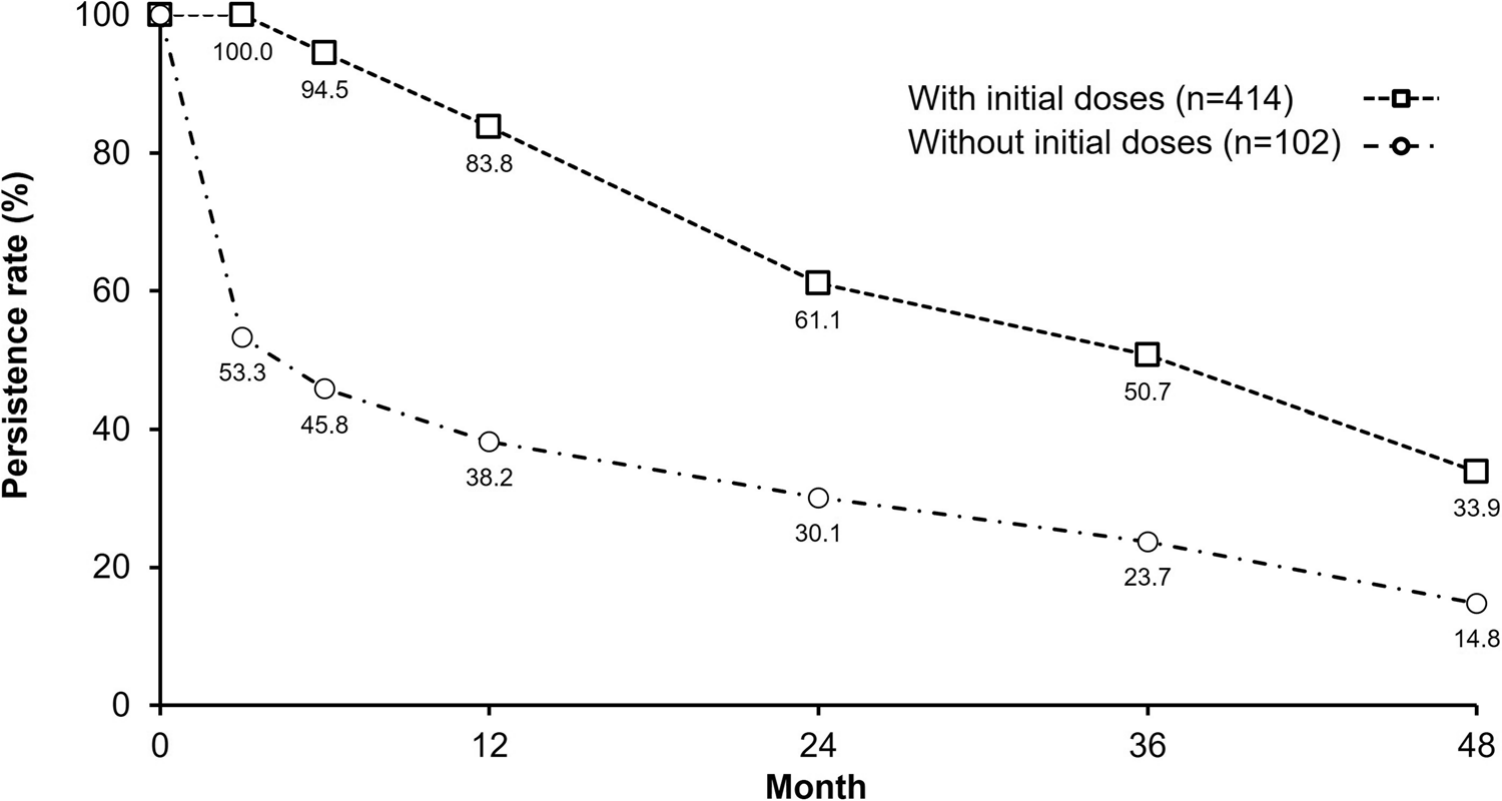
Estimated rate of persistence with intravitreal aflibercept up to year 4

A total of 414 of 516 patients (80.2%) in the FAS received the 3 initial injections within the first 45–90 days after treatment initiation (i.e., after the first injection in month 0). Notably, the early discontinuation rate was significantly higher (*p* < 0.0001) for patients who did not receive these initial 3 monthly injections of IVT-AFL than for those who did. By Month 3, 46.7% of patients who had not received the initial injections had discontinued IVT-AFL, whereas 100% of patients who had received the initial injections were still being treated (Fig. 1). By month 48, persistence with IVT-AFL treatment had decreased to 33.9% of patients who received initial dosing and 14.8% of those who did not.

In the overall FAS, the mean duration of follow-up after the first injection of IVT-AFL was 38.7 ± 15.3 months (median, 47.6 months); 306 patients (59.3%) were followed-up for ≥ 45 months. The duration of follow-up was defined as the time from study entry to the last visit performed, where the last visit did not necessarily need to involve a treatment and could have been a monitoring visit only.

During the follow-up period, 97 patients (18.8%) in the FAS discontinued because they switched from IVT-AFL and received another anti-VEGF agent or laser photocoagulation/photodynamic therapy and stopped IVT-AFL (or only resumed IVT-AFL > 3 months after the procedure). Most patients who switched treatment received another anti-VEGF, with 91/95 switching to ranibizumab; the main reason for switching was lack of efficacy (*n* = 66; 69.5%). More than half the patients who switched to another anti-VEGF (*n* = 55; 57.9%) later switched back to IVT-AFL.

The overall annualized rate of visits (mean [95% CI]) in the FAS was higher during the first year of treatment than in subsequent years (year 1, 9.83 [9.55–10.11]; year 2, 7.05 [6.81–7.31]; year 3, 6.70 [6.44–6.96]; year 4, 6.89 [6.61–7.18]). The overall annualized rate of injections in year 1 was 6.27 (95% CI, 6.05–6.50), and this rate decreased and remained relatively similar in subsequent years: 3.45 (3.28–3.63) in year 2, 3.20 (3.02–3.38) in year 3, and 3.35 (3.16–3.55) in year 4. There was no correlation between the annualized rate of injections in year 1 and BCVA score at baseline (data not shown). The number of injections and visits according to IVT-AFL treatment cohort is shown in Table 2.

**Table 2:** Mean number of visits and IVT-AFL injections from baseline to 12 months and 48 months for the overall FAS and each IVT-AFL treatment cohort

	12 months		48 months		
	IVT-AFL injections	Visits		IVT-AFL injections	Visits
FAS (*N* = 516)	6.0 ± 2.0	9.4 ± 2.3		13.3 ± 9.1	24.5 ± 11.4
Regular cohort (*n* = 102)	7.2 ± 0.8	9.4 ± 1.8		14.9 ± 7.4	23.6 ± 9.9
Irregular cohort with initial injections (*n* = 253)	6.2 ± 2.1	10.1 ± 1.9		13.7 ± 9.1	24.4 ± 9.3
Irregular cohort without initial injections (*n* = 56)	5.3 ± 1.7	8.7 ± 1.8		11.9 ± 7.4	22.7 ± 9.0

Values are mean ± SD. The regular and irregular cohorts include only those patients from the FAS with a BCVA assessment at baseline and month 12. *BCVA* best-corrected visual acuity, *FAS* full analysis set, *IVT-AFL* intravitreal aflibercept.

### Visual outcomes

Of the 516 patients in the overall FAS, 435 had a BCVA evaluation at baseline and month 12 and could be included in the analysis of the primary outcome. The overall mean change in BCVA (baseline, 57.2 ± 18.9) was 5.1 ± 15.7 letters at month 12 (*p* < 0.001). The change in mean BCVA between baseline and month 12 was higher in patients with a lower mean BCVA at baseline: 12.3 ± 18.7 letters (*p* < 0.001) in patients with < 50 letters (median baseline BCVA, 35.0); 8.5 ± 17.7 (*p* < 0.001) in patients with ≥ 50 to < 55 letters (median baseline BCVA, 50.0); 4.8 ± 13.8 (*p* < 0.001) in patients with ≥ 55 to < 70 letters (median baseline BCVA, 63.0); and − 1.4 ± 10.3 (*p* = not significant) in patients with ≥ 70 letters (median baseline BCVA, 75.0).

**Fig. 2:**
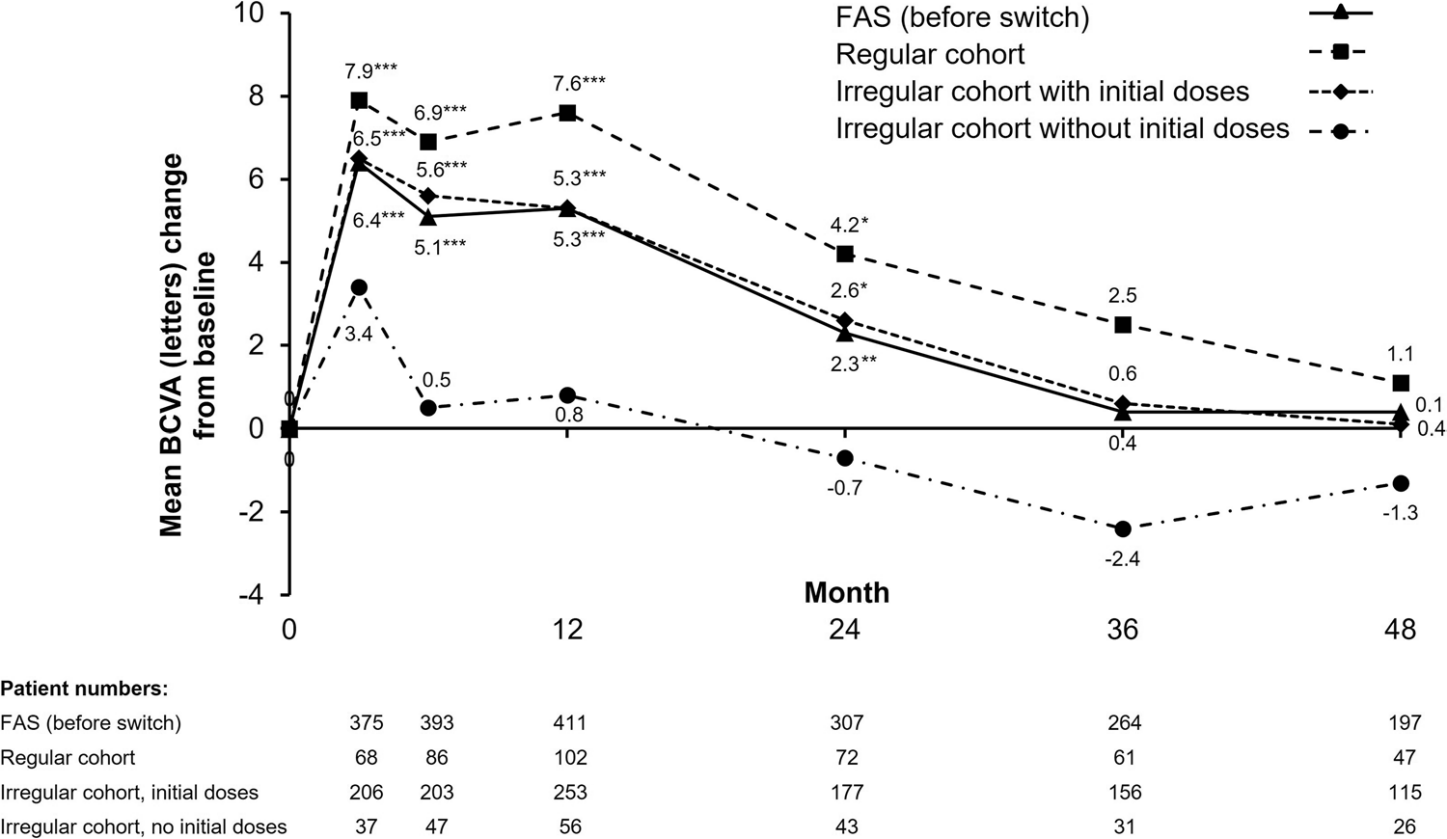
Mean change from baseline in BCVA for the FAS (before switch) and 3 IVT-AFL treatment cohorts. *p < 0.05, **p < 0.01, and ***p < 0.001 versus baseline (signed-rank test). BCVA, best-corrected visual acuity; FAS, full analysis set; IVT-AFL, intravitreal aflibercept

For all cohorts, the BCVA gains peaked after 3 months of treatment but returned to near-baseline levels by the end of the 48-month study period (Fig. 2). In the overall FAS, the mean BCVA was 56.5 ± 18.8 letters at baseline (*N* = 516), 62.6 ± 16.9 letters at month 3 (*n* = 376), 62.3 ± 19.1 letters at month 12 (*n* = 435), and 59.6 ± 22.3 letters at month 48 (*n* = 263). Change in BCVA from baseline was statistically significant for visits at months 3, 6, 12, and 24 (*p* < 0.001) in the overall FAS; however, no significant change was observed from baseline to month 36 or month 48.

There were no significant differences in the change in BCVA from baseline to month 48 between patients in the FAS who received < 7 or ≥ 7 injections in the first 12 months of treatment (data not shown). Similarly, there were no clear trends when considering the number of injections received (0–3, 4–6, 7–9, and > 9) during the first 12 months and the gain or loss of letters (Online Resource 2). However, patients who received 3 initial monthly IVT-AFL injections followed by regular treatment showed a trend of improved functional outcomes over 24 months (Online Resource 3) and 48 months (Fig. 2) compared with patients who received irregular treatment without the initial injections.

Of 263 patients in the FAS with a BCVA assessment at month 48, between baseline and month 48, 65 patients (24.7%) gained ≥ 15 letters, 88 (33.5%) gained ≥ 10 letters, 122 (46.4%) gained ≥ 5 letters, and 28 (10.6%) gained 0–4 letters, whereas 50 patients (19.0%) lost ≥ 15 letters. The proportion of these patients with BCVA < 50 letters was 24.3% (64/263) at baseline and 27.4% (72/263) at month 48. In the overall FAS, the proportion of patients with a BCVA ≥ 70 letters increased significantly (*p* = 0.010) from 35.7% (*n* = 94) to 45.6% (*n* = 120), with the proportion of patients achieving a BCVA ≥ 70 letters being numerically highest in the regular cohort (Online Resource 4).

### Anatomic outcomes

**Fig. 3:**
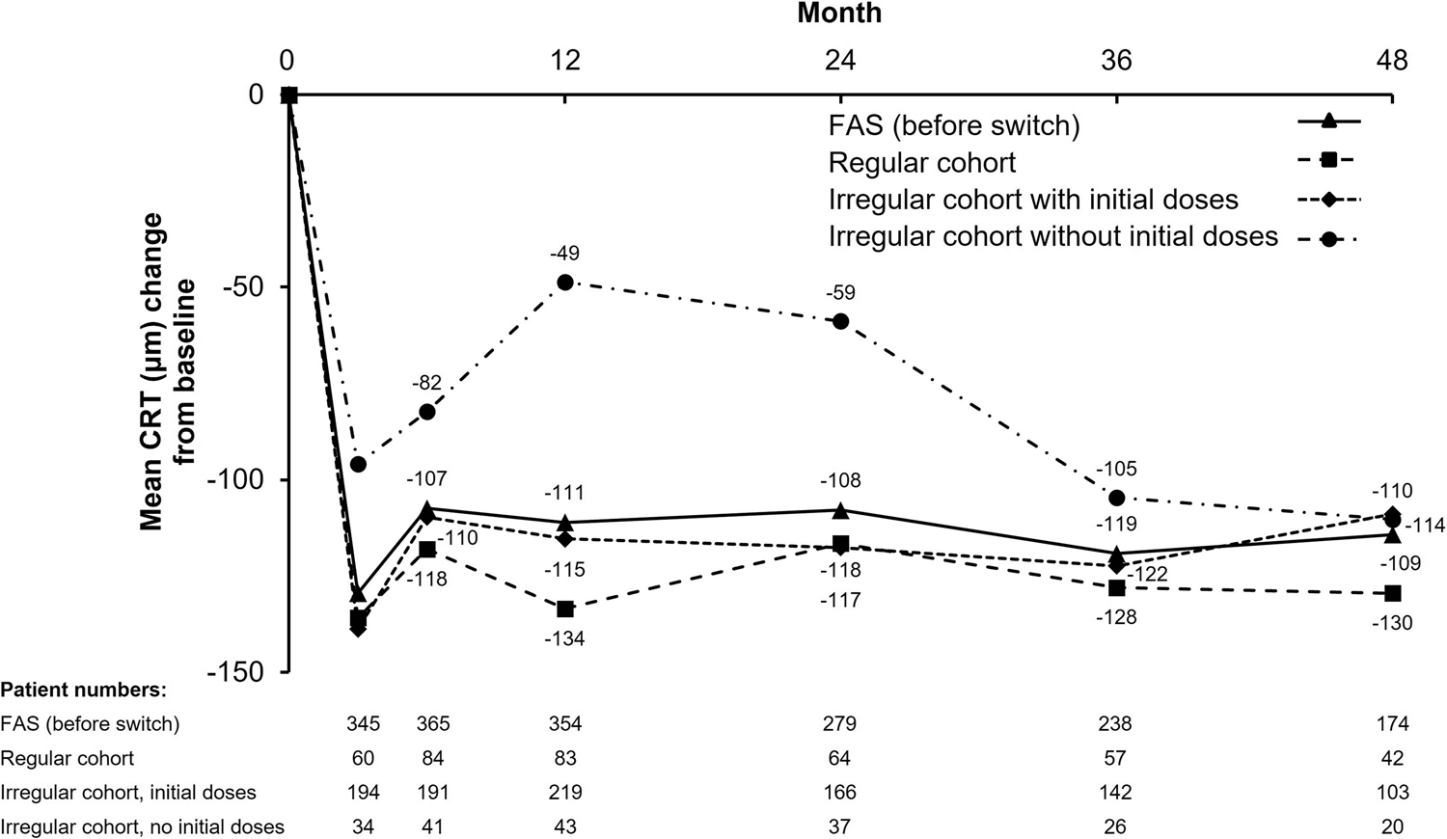
Mean change from baseline in CRT for the FAS (before switch) population and 3 IVT-AFL treatment cohorts. The mean change in CRT from baseline was significant for all time points and cohorts (*p* < 0.001; signed-rank test). CRT, central retinal thickness; FAS, full analysis set; IVT-AFL, intravitreal aflibercept

In the overall FAS population, mean CRT decreased markedly between baseline and month 3 from 400 ± 141 μm to 270 ± 81 μm (decrease of 130 ± 147 μm; *p* < 0.001). Changes from baseline remained statistically significant over the study period, including at month 48 (− 118 μm [95% CI, − 135 to − 101]; *p* < 0.001). At month 48, significant decreases in CRT were observed in all cohorts (Fig. 3).

### Safety outcomes

During treatment with IVT-AFL and until 30 days after the last injection, treatment-emergent adverse events (TEAEs) were reported in 280 of 591 patients (47.4%) (Table 3). TEAEs in 87 patients (14.7%), including lack of efficacy in 25 patients, were considered related to IVT-AFL treatment by the investigators. The most common serious TEAEs were cardiac failure (*n* = 6) and transient ischemic attack (*n* = 4). Five deaths occurred during the study (metastatic bronchial carcinoma, peritoneal metastases, and fatal fall [*n* = 1 each] or no additional details provided (*n* = 2)) and were considered unrelated to IVT-AFL treatment. The most common ocular TEAEs were lack of efficacy (*n* = 25, 4.2%) and serous retinal detachment (*n* = 19, 3.2%). Serious ocular TEAEs occurred in 14 of 591 patients (2.4%), of which uveitis and cataract were the most common (3 events in 2 patients each). Of the 14 patients with serious ocular TEAEs, one of the patients (with uveitis) discontinued IVT-AFL as the event was considered related to treatment. In terms of TEAEs related to intraocular inflammation, there were 4 cases of ulcerative keratitis (0.7%), 2 of punctate keratitis (0.3%), 1 of keratitis (0.2%), and 1 of eye inflammation (0.2%).

**Table 3:** IVT-AFL safety summary at 48 months

Number of patients (%)	Patients (*N* = 591)
Any TEAE	280 (47.4)
Ocular	187 (31.6)
Non-ocular	145 (24.5)
Any TEAE (treatment-related)	87 (14.7)
Ocular	80 (13.5)
Non-ocular	7 (1.2)
Most common ocular TEAE (> 0.5%)	
Lack of efficacy	25 (4.2)
Serous retinal detachment	19 (3.2)
Visual acuity reduced	16 (2.7)
Vitreous floaters	14 (2.4)
Lacrimation increased	12 (2.0)
Therapy change	11 (1.9)
Detachment of retinal pigment epithelium	10 (1.7)
Eye pain	9 (1.5)
Cataract	9 (1.5)
Metamorphopsia	9 (1.5)
Ocular hypertension	8 (1.4)
Retinal edema	7 (1.2)
Vision blurred	7 (1.2)
Blepharitis	6 (1.0)
Conjunctivitis	6 (1.0)
Conjunctival hemorrhage	5 (0.8)
Eye irritation	5 (0.8)
Eye pruritus	5 (0.8)
Photophobia	5 (0.8)
Retinal exudates	5 (0.8)
Retinal neovascularization	5 (0.8)
Choroidal neovascularization	4 (0.7)
Drug hypersensitivity	4 (0.7)
Dry eye	4 (0.7)
Inappropriate schedule of product administration	4 (0.7)
Injection site pain	4 (0.7)
Ulcerative keratitis	4 (0.7)
Visual field defect	4 (0.7)
Diplopia	3 (0.5)
Discontinuation due to TEAE	109 (18.4)
Discontinuation due to TEAE (treatment-related)	61 (10.3)
Any serious TEAE	85 (14.4)
Ocular	14 (2.4)
Non-ocular	74 (12.5)
Serious ocular TEAE	14 (2.4)
Cataract	2 (0.3)
Retinal detachment	2 (0.3)
Subretinal hematoma	2 (0.3)
Uveitis	2 (0.3)
Vitreous hemorrhage	2 (0.3)
Conjunctival hemorrhage	1 (0.2)
Retinal artery occlusion	1 (0.2)
Retinal hemorrhage	1 (0.2)
Retinal vein occlusion	1 (0.2)
Traumatic cataract	1 (0.2)
Visual field defect	1 (0.2)

*AE* adverse event, *IVT-AFL* intravitreal aflibercept, *TEAE* treatment-emergent adverse event.

## Discussion

In RAINBOW, treatment-naïve patients with nAMD treated with IVT-AFL in routine clinical practice in France maintained anatomic improvements over 4 years, with a mean change in CRT of − 118 μm at month 48 for the overall FAS. However, the initial mean gain in BCVA of + 5.1 letters at month 12 (baseline, 57.2 letters) was not maintained, with visual acuity returning toward baseline levels (and below baseline levels in patients who received the fewest number of injections, namely, the irregular treatment cohort without the 3 initial injections) by the end of the study because of progressive undertreatment.

Although more than 80% of patients received the 3 initial monthly injections of IVT-AFL, the majority of patients were not treated regularly over the full study period. Those patients who did receive the 3 initial monthly injections showed greater persistence with treatment, and those who were treated regularly during the first year experienced better functional outcomes than patients who received irregular treatment without the initial injections. However, the 4-year outcomes were similar for all treatment cohorts, suggesting an association with undertreatment after the first year. Based on the data, we cannot draw any conclusions regarding whether the improved outcomes in patients with regular treatment were due to treatment frequency or whether treatment frequency was higher in these patients because they experienced better outcomes and were, therefore, more likely to adhere to treatment. The mean number of visits was higher than the mean number of IVT-AFL injections received, implying that more patients may have been treated pro re nata instead of by T&E. The overall safety profile of IVT-AFL was consistent with previous studies.

The BCVA gains observed in RAINBOW are lower than those observed in the key RCTs of IVT-AFL treatment of nAMD: in the VIEW 1 and 2 studies, patients who received 3 initial monthly injections and treatment every 2 months thereafter had a mean gain of + 7.6 letters (baseline, 53.6 letters) over 96 weeks, after a mean of 11.2 injections [3].

The 12-month BCVA gains in RAINBOW are numerically higher, however, than those observed in earlier real-world studies of anti-VEGF treatment of nAMD in France. In LUMIERE, a mean gain of + 3.2 letters from baseline was observed after 12 months of ranibizumab treatment in 551 patients; < 40% of patients received the recommended initial injections, and patients received a mean of 5.1 injections over 12 months [8]. In TWIN, a follow-up study incorporating many of the centers involved in LUMIERE, a slight improvement was observed in the mean visual acuity gain (+ 4.3 letters) after 12 months of ranibizumab treatment in 881 patients; 56.6% of these patients received the 3 initial injections, with a mean of 5.6 injections over 12 months [9]. In the AURA study performed in 8 countries, including France, treatment with ranibizumab in the French cohort resulted in only a + 0.8 letter gain at 12 months from baseline (56.0 letters), dropping to a mean change of − 1.1 letters after 2 years of treatment; patients received a mean of just 6.3 injections over 2 years and only 53% of patients completed 2 years of follow-up [10].

Other European real-world studies of IVT-AFL treatment further highlight the challenges inherent in longer-term, non-interventional studies with respect to study discontinuations, low treatment persistence, and irregular treatment. In the German PERSEUS study, treatment-naïve patients who received regular IVT-AFL treatment achieved a mean visual acuity gain of + 8.0 letters (baseline, 52.8 letters) compared with + 4.0 letters (baseline, 53.7 letters) among those who received irregular treatment in year 1 [15], and the trends observed after 2 years of treatment were similar to those observed in year 1 (+ 6.3 letters in the regular cohort and + 3.3 letters in the irregular cohort (free LOCF populations)) [16]. Patients received a mean of 8.0 injections over 24 months (regular, 13.1; irregular, 7.8), and only 28.0% and 6.5% of patients had received regular treatment by the end of year 1 and year 2, respectively (defined as IVT-AFL injections every 2 months after 3 initial monthly injections, with ≥ 7 injections in year 1 and ≥ 4 injections in year 2). Approximately 62% of the original 803-patient cohort had discontinued the study by month 24.

In a retrospective single-center case series analysis of IVT-AFL treatment of nAMD based in the UK, a mean gain of + 5.9 letters at 1 year, + 6.4 letters at 2 years, and + 6.6 letters at 3 years from a baseline of 54.4 letters was reported [17]. The initial gains were achieved and maintained with regular treatment, consisting of a mean total of 7.2, 12.0, and 15.9 injections over each time period, respectively. Despite these promising outcomes, almost one-third of patients did not complete 3 years of follow-up. Furthermore, a retrospective study by the UK Aflibercept Users Group (using electronic medical records from 1083 patients over 2 years) similarly found that more regular IVT-AFL treatment was associated with improved visual outcomes [18].

To date, the most extensive real-world data on the anti-VEGF treatment of nAMD have been collected by FRB!. The first 10-year outcome report based on FRB! data found that patients in Australia and New Zealand who completed 10 years of continuous treatment lost a mean of only 0.9 letters, whereas those in Switzerland lost a mean of 14.9 letters [19]. Notably, the median number of injections in the Australian and New Zealand cohorts was higher than that in the Swiss cohort (53 vs 42 injections over 10 years). A second report on 10-year outcomes using FRB! data from France identified a loss of 18 letters after a median of only 27.5 injections over 10 years [11]. Other 10-year follow-up studies in Australia and the UK have similarly suggested that vision can be maintained at 10 years if treatment is sufficiently regular [20, 21].

Thus, the findings of RAINBOW are consistent with those of prior RWE, emphasizing the importance of 3 initial monthly IVT-AFL injections followed by continuous proactive treatment beyond the first year. In routine clinical practice, the frequency of anti-VEGF treatment of nAMD tends to be less regular, which can lead to poorer visual outcomes compared with RCTs [5]. This is in part due to the burden of regular treatment, particularly on older patients, who comprise the majority of the population with nAMD. Recognition of the challenges associated with treatment burden has led to a shift toward more personalized treatment regimens, such as pro re nata and T&E, with the aim of reducing treatment frequency without sacrificing gains in visual acuity. However, the relative undertreatment of patients persists in routine clinical practice and is associated with progressive vision loss. The risk factors associated with non-adherence to and non-persistence with treatment must be further investigated to promote improved patient care and outcomes in nAMD [22].

Besides loss to follow-up, several other limitations inherent to the observational design of RAINBOW may affect interpretation of the findings reported here. A variety of charts were used to assess visual acuity, and this may have introduced bias. Furthermore, the scheduling of patient visits and monitoring was at the discretion of the attending physician, and this led to a large quantity of missing data.

## Conclusions

This 48-month analysis of the RAINBOW study of the real-world use of IVT-AFL in France to treat nAMD demonstrated a lack of long-term effectiveness due to undertreatment and poor persistence. The treatment frequency for all cohorts, including the regular cohort, was at the discretion of the treating physician, and progressive undertreatment after the first year resulted in the loss of initial vision gains. Treatment-naïve patients who received 3 initial monthly IVT-AFL injections followed by regular treatment over the first 12 months showed a trend of improved functional outcomes over 48 months compared with patients who received irregular treatment without the initial injections. Further, patients who received the 3 initial monthly IVT-AFL injections were much more likely to persist with treatment than those who did not. Arguably, patients are more likely to persist with treatment when they are achieving positive outcomes, and patients are more likely to achieve positive outcomes if they persist with treatment; we cannot definitively separate these effects. Overall, the initial monthly injections followed by continuous proactive treatment beyond the first year appear to be key to achieving optimal real-world functional outcomes with IVT-AFL in patients with nAMD. Future studies need to examine the factors underlying poor persistence and undertreatment and explore ways in which to promote regular treatment in routine clinical practice in France. For example, patient education to manage expectations may be necessary, to emphasize that regular treatment is needed to achieve improved outcomes.

## Supplementary Information

Below is the link to the electronic supplementary material.

Supplementary file1 (PDF 221 KB)

## Data Availability

Availability of the data underlying this publication will be determined later according to Bayer’s commitment to the EFPIA/PhRMA “Principles for responsible clinical trial data sharing.” This pertains to scope, time point, and process of data access. As such, Bayer commits to sharing upon request from qualified scientific and medical researchers patient-level clinical trial data, study-level clinical trial data, and protocols from clinical trials in patients for medicines and indications approved in the United States (US) and European Union (EU) as necessary for conducting legitimate research. This applies to data on new medicines and indications that have been approved by the EU and US regulatory agencies on or after January 01, 2014. Interested researchers can use www.clinicalstudydatarequest.com to request access to anonymized patient-level data and supporting documents from clinical studies to conduct further research that can help advance medical science or improve patient care. Information on the Bayer criteria for listing studies and other relevant information is provided in the study sponsors section of the portal. Data access will be granted to anonymized patient-level data, protocols, and clinical study reports after approval by an independent scientific review panel. Bayer is not involved in the decisions made by the independent review panel. Bayer will take all necessary measures to ensure that patient privacy is safeguarded.
