# Supplementary material for: Final 4-year results of the RAINBOW real-world study: intravitreal aflibercept dosing regimens in France in treatment-naïve patients with neovascular age-related macular degeneration
Source: Graefes Arch Clin Exp Ophthalmol. 2022 Nov 18;261(4):959–69. doi: 10.1007/s00417-022-05900-6 (PMC10049954; doi:10.1007/s00417-022-05900-6)

Supplementary Information

Final 4-year results of the RAINBOW real-world study: Intravitreal aflibercept dosing regimens in France in treatment-naïve patients with neovascular age-related macular degeneration

Salomon-Yves Cohen,[1] Marcel Dominguez,[2] Florence Coscas,[3] Céline Faure,[4] Stéphanie Baillif,[5] Hassiba Oubraham,[6] Laurent Kodjikian,[7] Michel Weber[8]; on behalf of the RAINBOW study investigators

[1]Centre d'Imagerie et de Laser, Paris, France; Department of Ophthalmology, University Paris-Est Créteil, Paris, France
[2]Centre Rétine Gallien, Bordeaux, France
[3]Centre Ophtalmologique de l'Odéon, Paris, France
[4]Hôpital privé Saint Martin, Ramsay Générale de Santé, Caen, France
[5]Pasteur 2 Teaching Hospital, Université Côte d'Azur, Nice, France
[6]Centre OPHTA-45, Montargis, France
[7]Croix-Rousse University Hospital, Hospices Civils de Lyon, University of Lyon I, Lyon, France; CNRS 510 MATEIS INSA Lyon, Université de Lyon Claude Bernard, Villeurbanne, France
[8]CHU Hôtel-Dieu, Nantes, France

Corresponding author:
Dr. Salomon-Yves Cohen
C.I.L. 11 Rue A. Bourdelle
Paris, France
Tel: +33 1 42 84 94 00
Email: sycsyc75@gmail.com
ORCID iD: 0000-0003-1181-4379

Online Resource 1 Patient disposition. BCVA, best-corrected visual acuity; FAS, full analysis set; IVT-AFL, intravitreal aflibercept; SAS, safety analysis set.

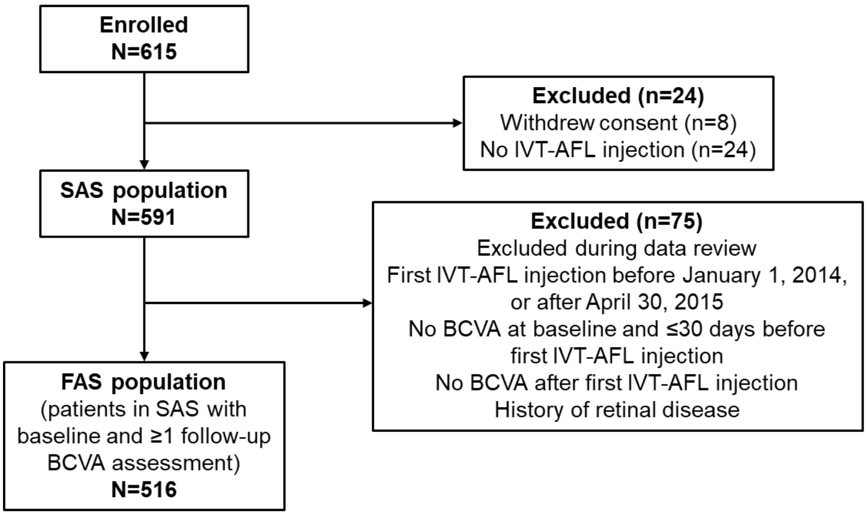

Online Resource 2 Gain or loss of letters in the FAS at Month 48 stratified by the number of IVT-AFL injections received between Month 0 and Month 12. FAS, full analysis set; IVT-AFL, intravitreal aflibercept.

No. of patients (%)	0–3 injections (n=29)	4–6 injections (n=85)	7–9 injections (n=135)	>9 injections (n=14)
Gain of 0–4 letters	4 (13.8)	10 (11.8)	13 (9.6)	1 (7.1)
Gain of 5–9 letters	3 (10.3)	8 (9.4)	21 (15.6)	2 (14.3)
Gain of 10–14 letters	3 (10.3)	5 (5.9)	12 (8.9)	3 (21.4)
Gain of ≥15 letters	8 (27.6)	26 (30.6)	27 (20.0)	4 (28.6)
Loss of ≤15 letters	4 (13.8)	15 (17.6)	35 (25.9)	3 (21.4)
Loss of >15 letters	7 (24.1)	21 (24.7)	27 (20.0)	1 (7.1)

Online Resource 3 Mean change in BCVA from baseline to Month 24 for the 3 treatment cohorts, stratified by the number of IVT-AFL injections received between Month 12 and Month 24 (<4 or ≥4 injections). BCVA, best-corrected visual acuity; IVT-AFL, intravitreal aflibercept; M, month.

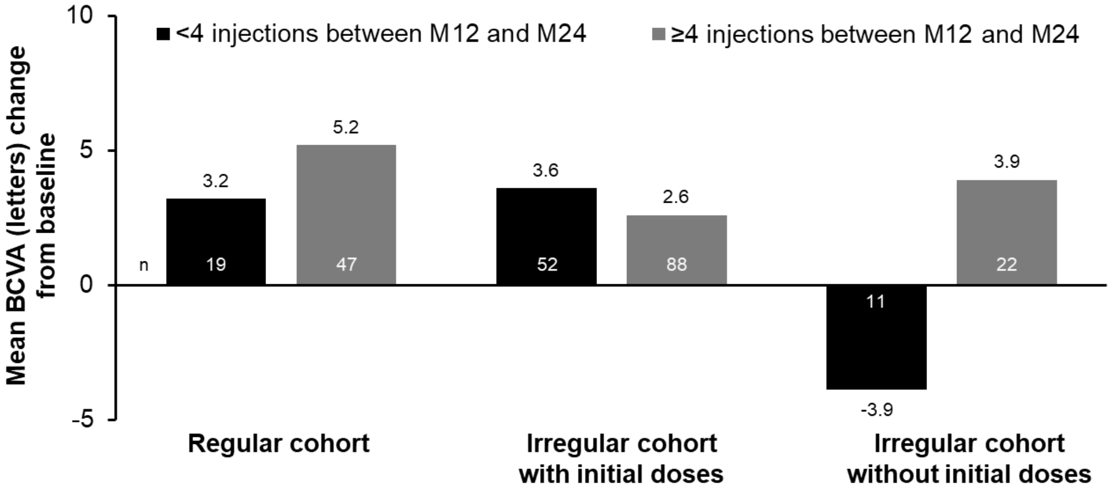

Online Resource 4 Proportion of patients achieving a BCVA ≥70 letters for the FAS (before switch) and 3 IVT-AFL treatment cohorts. BCVA, best-corrected visual acuity; FAS, full analysis set; IVT-AFL, intravitreal aflibercept.

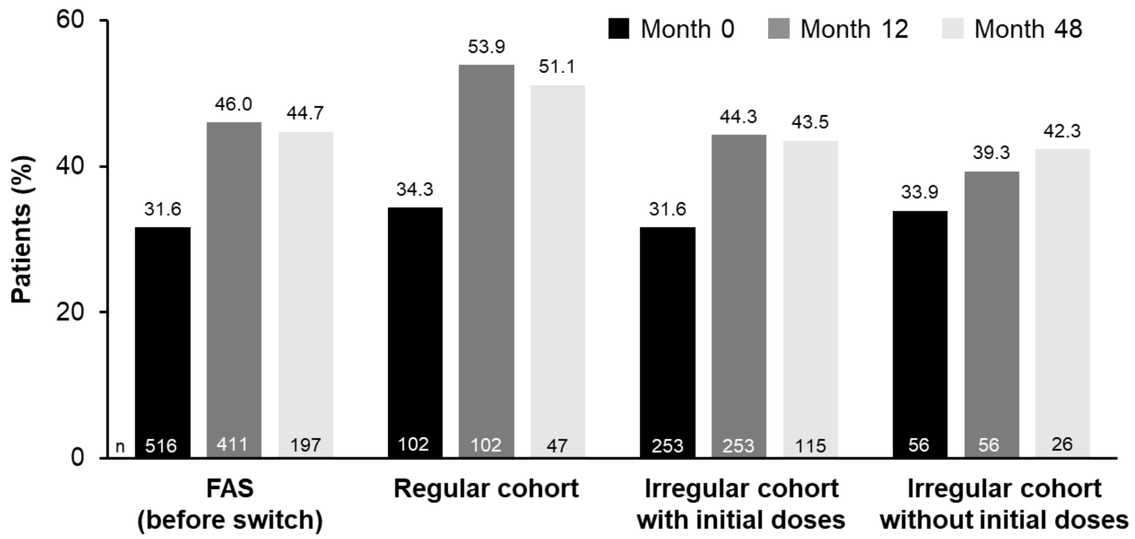

Supplement: Supplementary file 1 — Supplementary file1 (PDF 221 KB) [file 417_2022_5900_MOESM1_ESM.pdf]
